# Pluripotent Stem Cell-derived Dopaminergic Neurons for Studying Developmental Neurotoxicity

**DOI:** 10.1007/s12015-023-10555-9

**Published:** 2023-06-05

**Authors:** Anna Kreutz, Guang Hu, Erik Tokar

**Affiliations:** 1 Mechanistic Toxicology Branch, Division of Translational Toxicology, NIEHS, Research Triangle Park, Durham, NC 27709, USA; 2 Epigenetics and Stem Cell Biology Laboratory, Division of Intramural Research, NIEHS, Research Triangle Park, Durham, NC 27709, USA

**Keywords:** Developmental neurotoxicity, Pluripotent stem cells, Dopaminergic neurons, Risk assessment, Environmental health, New approach methodologies

## Abstract

With the vast number of chemicals in commerce, higher throughput strategies are needed to inform risk assessment. The field of toxicology is therefore moving away from traditional *in vivo* guideline studies towards *in vitro* new approach methodologies. There has been a great push for such a shift in the field of developmental neurotoxicity, where there is a particular lack of data. A battery of *in vitro* new approach methodologies has thus been developed to help fill this gap. Included in this battery are assays for numerous processes critical to neurodevelopment, such as proliferation, migration, and synaptogenesis. The current battery of developmental neurotoxicity new approach methodologies still lacks recapitulation of several critical neurodevelopmental processes, including development of neuronal subtypes. With their pluripotency, alongside other advantages, pluripotent stem cells (PSCs) are uniquely suited to address questions of developmental neurotoxicity, as they can recapitulate the different stages of human *in vivo* neurodevelopment. Among the various neuronal subtypes, development of dopaminergic neurons (DA) is perhaps the best understood and several approaches exist to differentiate PSCs into DA. Herein we review these approaches and propose utilizing PSCs for screening of the impact of environmental chemicals on development of DA. Related techniques and gaps in knowledge are also addressed.

## Introduction

The discovery of stem cells has opened great promise in numerous arenas for both research and therapeutic applications. Uptake in the field of toxicity testing, however, has been relatively slow. With the shift away from animal studies due to their lack of recapitulation of human biology, costs, and concerns of animal welfare, *in vitro* cell-based assays are increasingly being employed for toxicity testing [4, 41].

The push to move away from *in vivo* behavioral and endpoint assessments to *in vitro* assays is especially strong in the field of developmental neurotoxicity (DNT). This stems from many factors, but particularly the major dearth of data for DNT. This gap has come to the fore with the rise in diagnoses of children with neurodevelopmental disorders and concerns that chemical exposure may be contributing to this increasing incidence [6, 26]. Testing for DNT is not a regulatory requirement and is highly resource intensive; accordingly, DNT guideline studies have been performed on less than 1% of the ~ 75,000 chemicals in commerce [4, 30]. A battery of *in vitro* new approach methodologies (NAMs) for DNT is being established to help address this data gap [4, 7, 41]. This DNT battery can be performed in mid to high-throughput and assesses a range of morphological and functional endpoints, including proliferation, neurite outgrowth, and network activity, which can provide mode of action information [11]. The utility of DNT NAMs for risk assessment is beginning to be realized, with data from DNT NAMs recently used in waiving the need for a guideline DNT study [21]. The current DNT NAM battery covers a broad range of processes and endpoints critical to human neurodevelopment that could be disrupted and thereby contribute to DNT, such as migration and neural network formation, as outlined in the graphical abstract. The combination of these numerous assays can allow for distinguishing the mechanisms underlying compound toxicity, as well as windows of susceptibility. However, this battery still lacks several key features of brain development, including recapitulation of neuronal subtype development.

A variety of cell types are used in DNT NAMs from both rodents and human, including cell lines, primary cells, and neural precursor cells (NPCs). Human pluripotent stem cells (hPSCs), which, in the context of this review, encompass both embryonic stem cells (ESCs) and induced PSCs (iPSCs), are particularly well suited for DNT NAMs due to their pluripotency, differentiation potential, ability to be expanded for large-scale studies, and recapitulation of human *in vivo* development [35]. Moreover, they can be readily manipulated with biochemical, genetic, and genomic approaches, and routine culture techniques have now been established for them.

Protocols have been established to differentiate PSCs into a variety of neuronal lineages, including cholinergic, dopaminergic (DA), glutamatergic, and GABAergic [12, 27, 35, 48, 53]. The mechanisms driving lineage differentiation are perhaps best understood for DA and multiple approaches can be used to generate relatively pure cultures of DA [12, 35, 47]. Moreover, the gene expression of hPSC-derived midbrain DA is similar to *in vivo* [3].

While DA constitute less than 1% of neurons in the brain, they play critical roles in stress, mood, movement, reward, and addiction. The rate limiting enzyme for DA synthesis is tyrosine hydroxylase (TH), which is commonly used as a marker for DA, along with the vesicular monoamine transporter (VMAT2) and DA active transporter (DAT). DA are located in the diencephalon, mesencephalon, and olfactory bulb, with 90% found in the midbrain—specifically the substantia nigra pars compacta (SNpc)—A9 neurons, ventrotegmental area (VTA)—A10 neurons, and retrorubral field (RRF)—A8 neurons [13]. A9 neurons give rise to the mesostriatal (or nigrostriatal) pathway, which contributes to motor function, while A10 neurons give rise to the mesolimbic pathway, involved in reward and emotions, and the mesocortical pathway, involved in reward and cognition [8]. The presence of DA in the SNpc is particularly well characterized as these are the neurons that are selectively degenerated in Parkinson’s Disease (PD) [40]. Likely related to their wide-ranging effects and broad distribution, DA have been implicated in a variety of neurodevelopmental and neurodegenerative disorders, with the linkage between DA and both neurodevelopmental and neurodegenerative disorders appearing to be related to the particular susceptibility of DA to environmental toxicants [10, 13, 33, 51]. An extensive body of research has been conducted on DA in PD, including potential treatment strategies. Much of this work has been conducted using PSC-derived DA, with several groups performing in vivo transplantation with these neurons to rescue the effects of PD, including in clinical trials [5, 35, 36, 61, 65]. Additional work is needed to address the impact of environmental chemicals on DA development. This may help to address the etiology of both the 90–95% of PD cases that are idiopathic, as well as other neurodevelopmental and neurodegenerative disorders, such as ADHD, autism spectrum disorders, and schizophrenia, whose risk genes include those involved in DA signaling [31, 33, 64].

## Methods for PSC Differentiation into DA

Differentiation of PSCs into DA *in vivo* begins with neuronal specification. Protocols were first developed for mouse cells and have since been adapted for hPSCs by extending the timing [60]. Stromal feeder cells are commonly used to induce neuroectodermal fate as this was both efficient and reproducible. One of the primary limitations with neural induction protocols has been the heterogeneity of cells derived, both in terms of differentiation state and subtype. As with all PSC protocols, care must be taken to ensure purity of cultures.

Now, the most commonly employed method of producing neural stem cells (NSCs) is through dual SMAD inhibition (dSMADi) [12]. The SMAD signaling pathway regulates numerous processes, including proliferation, differentiation, and apoptosis [60]. dSMADi produces a more homogeneous neuronal population than other methods and does not require a neural feeder layer or formation of embryoid bodies as prior methods. The basis for dSMADi is through inhibition of two signaling pathways that converge on SMAD—BMP and TGFβ—using small molecules [60]. The most commonly employed small molecules, largely for their potency, are LDN193189 and SB431542. LDN193189 inhibits BMP type I receptor signaling, while SB431542 blocks nodal and activin, and subsequently TGFβ, both of which converge on SMAD. dSMADi suppresses pluripotency and promotes neural differentiation, producing neural rosettes of NSCs within 11 days.

**Fig. 1:**
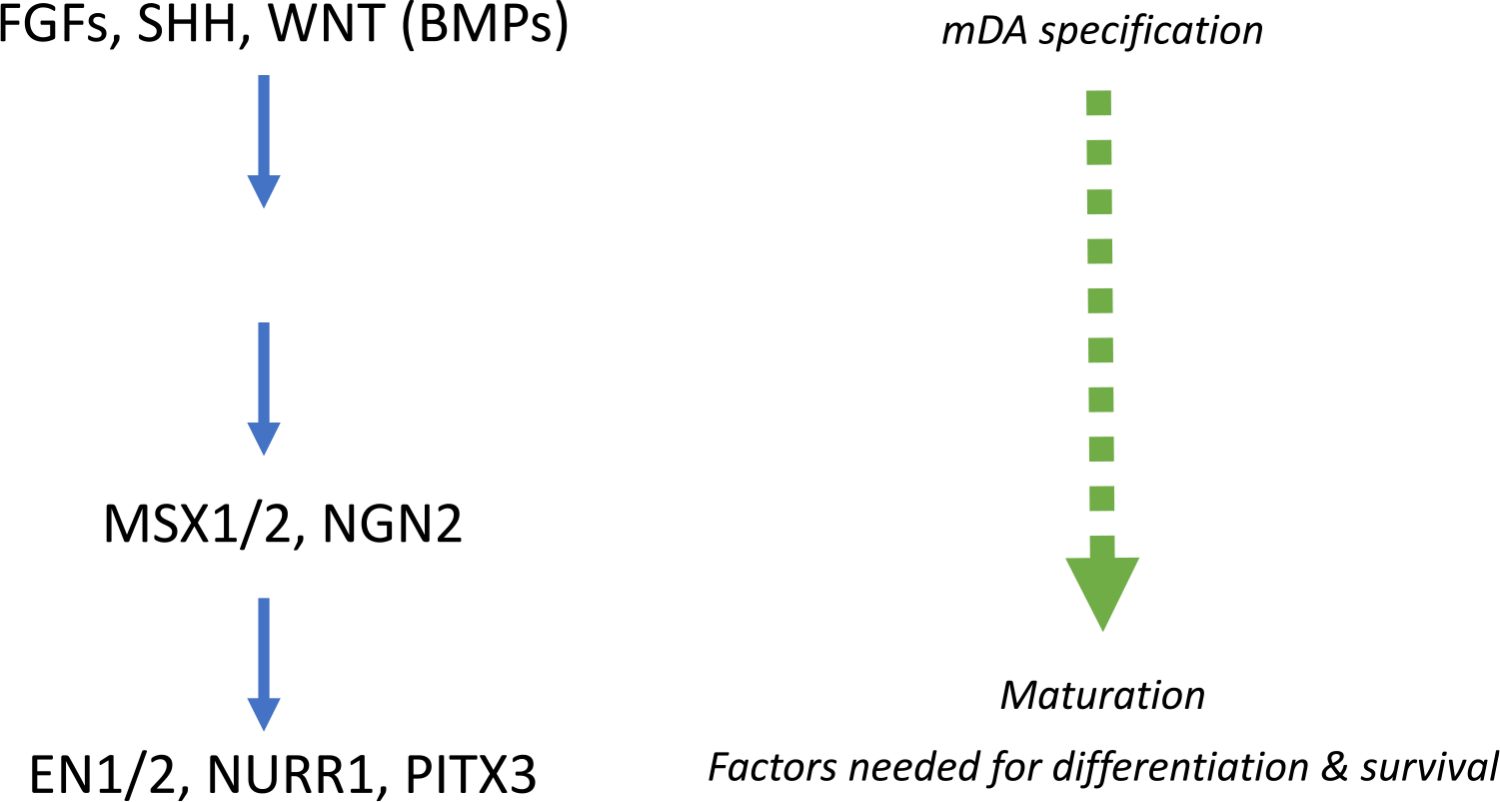
Signaling factors involved in DA development

PSCs can be subsequently differentiated into distinct neuronal subtypes. Most protocols for PSC differentiation into DA focus on production of midbrain DA (mDA), which comprise 90% of all DA cells in the brain. mDA differentiation begins ~E10.5–12 in the mouse, or 6–8.5 gestation weeks (GW) in humans [8, 60]. mDA are derived from the midbrain floor plate (FP) *in vivo*. The FP is found along the ventral midline of the neural tube, with mDA arising specifically from radial glia of the midbrain FP region [3]. The FP, in conjunction with the isthmic organizer, found at the midbrain/hindbrain boundary, confers regionalization through secretion of critical signaling factors. The combined actions of these signaling factors contribute to DA lineage specification (Fig. 1). *In vivo*, DA lineage specification is directed largely by sonic hedgehog (SHH), FGF8, and WNT1, which are necessary for mDA development [12, 60]. FGF8 and WNT1 are secreted from the isthmic organizer, driving OTX2 expression rostrally and GBX2 caudally [8]. The rostral midbrain FP expresses EN1 and DBX2.

**Fig. 2:**
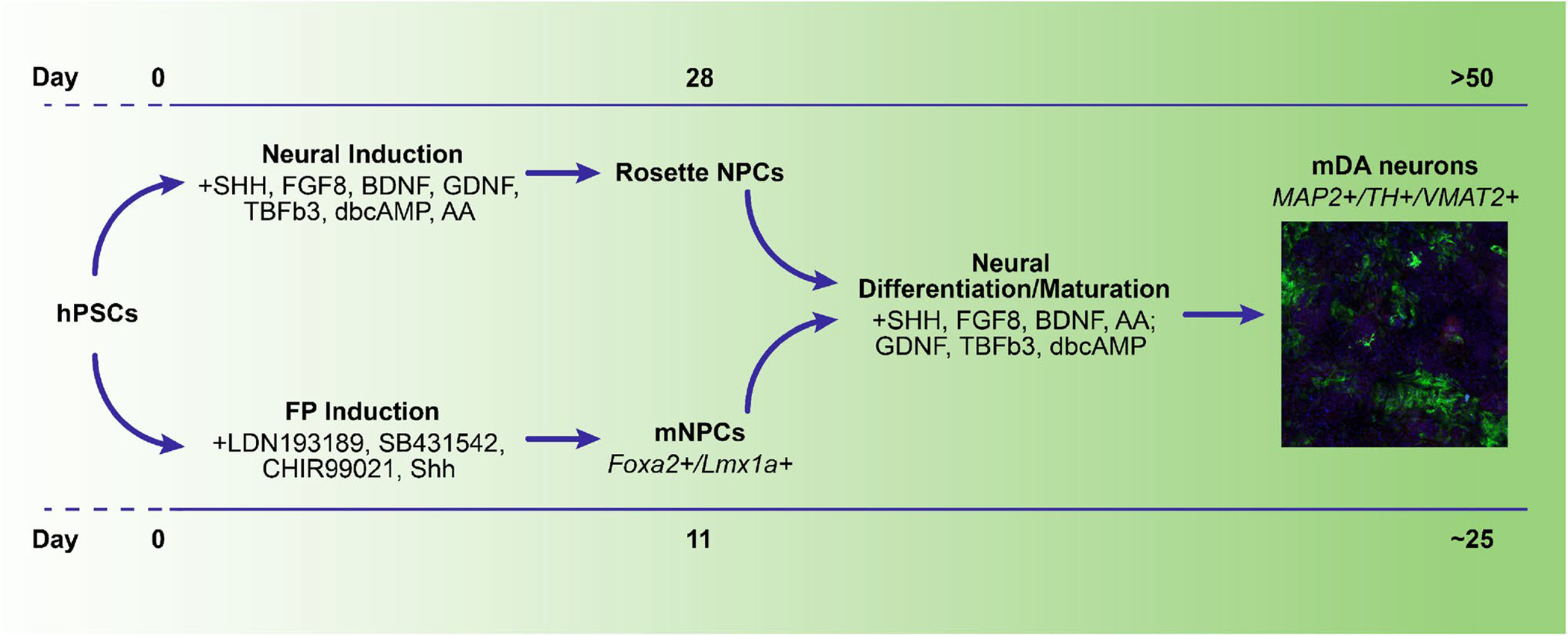
Methods of mDA production. hPSCs can be differentiated into mDA via two major approaches—through neural rosettes or through generation of a FP intermediate

Mediolateral regionalization, and mDA specification, is driven from the FP by WNT1 [8]. Once specified, mDA express FOXA2 and LMX1A—this expression is unique to the ventral midbrain, not being seen elsewhere in the central nervous system [60]. mDA FOXA2 + /LMX1A + expression is retained following maturation [3]. LMX1A expression extends somewhat more laterally than FOXA2 and is also a marker of the roof plate. FOXA2 plays a critical role in DA maturation—inducing Neurogenin2, leading to NURR1 and EN1 induction, markers needed for DA maturation [52]. Cells expressing TH, VMAT2, and DAT are commonly considered to be mature mDA—though additional functional confirmation is preferred. Further details of the various signaling factors involved in DA development are described in Table 1. mDA can be derived from PSCs, mesenchymal stem cells, and midbrain NPCs, and even directly from human dermal fibroblasts, as was recently shown [23, 60]. The first protocol that successfully derived DA, and a neuronal subtype more broadly, from hPSCs, was achieved via neural rosettes—neuroepithelial-like structures more commonly found in human than mouse [47]. In this approach, stromal feeder cells were used to induce neuroectodermal fate, with subsequent specification towards mDA identity using midbrain patterning molecules. Following differentiation, these committed DA precursors were matured into MAP2 + /TH + /AADC + /VMAT2 + neurons in 50 days (Fig. 2). This approach yielded up to 79% DA identity in the Tuj1 + neuronal population, based on TH expression, with only a small percentage of serotonergic and GABAergic neurons, though these mDA were relatively immature.

Later, a more rapid, higher purity, method for mDA production from hPSCs was achieved by combining dSMADi and FP production [35]. In this approach, midbrain precursors were produced without need for stromal feeder cells or formation of neural rosettes.

Production of DA through a FP intermediate was first engineered by the Studer lab [35]. The protocol relies on SHH to suppress an anterior fate, along with CHIR99021, an inhibitor of GSK3β, that thereby activates WNT1. SHH signaling is efficiently activated with purmorphamine, a small molecule agonist, either with or without recombinant SHH. Throughout the 11-day protocol, the basal media is phased from knockout serum replacement to neurobasal with B27, producing > 70% FOXA2 + /LMX1A + mDA precursors. These precursors can then be further differentiated into TH + mDA in 25 days. Gene expression analyses show generated mDA reflect A9 and A10 DA of the SN and VTA, respectively [60]. In comparison to mDA production that follows neural induction via rosette formation, the FP protocol produces a higher yield of TH + cells with greater DA expression and few GABA or serotonergic neurons, which are commonly produced during neuronal differentiation. This FP protocol continues to be the most common method for DA production. The FP approach has been refined to yield > 90% LMX1A + /FOXA2 + cells, with minimal PAX6 expression, a marker of dorsal forebrain precursors, by day 11 and successfully employed with various human induced PSC (hiPSC) lines [17, 18, 24]. The small molecules used are fairly standardized, with only slight modifications, such as A83 instead of SB431542, or CT99021 instead of CHIR [8, 22]. While most protocols still begin with the knockout serum replacement basal medium, serum-free, defined media versions have also been established [16, 44]. Due to the widespread use of this method, kits are now available from various companies to produce mDA, with these being serum-free, defined media. The protocol has also been modified for 3D, including for use of embryoid bodies and organoid production [9, 22, 57]. Inclusion of an intermediate step through sphere formation has helped to increase yield and specificity.

An alternative approach to production of a FP mDA precursor for generation of DA is through overexpression or transduction of key signaling factors. Perhaps the most common overexpression approach for mDA is using NURR1—a transcription factor expressed highest in the SN, VTA, and RRF of the midbrain and limbic systems [52]. *In vivo,* NURR1 activates the TH promoter and is necessary for *Dat* and *Vmat* induction, being also suggested to regulate *Pitx3*—a regulator of TH. Production of mDA using NURR1 overexpression has been applied either alone or in conjunction with other factors such as *Foxa2*, *Neurogenin2*, *Ascl1*, or *Pitx3*. Mixed results have been found with addition of these other factors, with *Foxa2* and *Ascl1* showing the most consistent increase in mDA yield. A recent study obtained a relatively high proportion of induced DA from human dermal fibroblasts after testing several combinations of reprogramming factors, finding *Ascl1* with *Lmx1a, Lmx1b*, *Foxa2*, *Otx2*, and *Nr4a2* to be the most efficient [23]. While overexpression approaches are faster, easier, and cheaper, they do not recapitulate normal development, which may be critical for understanding healthy neurodevelopment and DNT.

Co-culturing of DA with other cell types allows for identification of cell-autonomous versus non-cell autonomous effects, better approximating *in vivo* cell states and interactions. Several groups have established approaches to culture DA with other cell types, particularly glia, as both astrocytes and microglia are known to play critical roles in neurodevelopment and neurogenesis [9, 17, 18, 34, 55]. These cultures can be set up as direct or indirect cultures to tease apart contributions of their respective effects as opposed to secreted factors. Moreover, the ability to readily perform genetic manipulations of hiPSCs allows for looking at the contribution of genetic factors in both a cell-specific manner and in combination. Co-culture approaches do have their limitations as they are more difficult to reliably establish and often introduce additional sources of variability.

Astrocytes provide important trophic support for neurons and dysfunctional astrocytes can mediate neurodegeneration. Astrocytes can be cultured with mDA, either at the precursor stage, or once matured [9, 17, 18, 34]. As both neurons and astrocytes arise from NPCs, both cell types can be produced from the same set of NPCs. Such an approach has been developed to produce midbrain FP precursors, followed by subsequent differentiation to mature astrocytes and mDA, independently. These studies have shown the important protective and damaging roles astrocytes play. While a cultured line of DA alone resulted in ferroptosis-induced programmed cell death, co-culture with astrocytes rescued this effect in DA [9, 34]. Moreover, this approach has been used to study the effects of specific mutations, such as in PD risk genes, on mDA. In one study, the impact of a PD-related mutation in LRRK2, one of the most common PD risk genes, in both mDA and astrocytes was assessed [18]. This mutation was found to alter production of extracellular vesicles and multivesicular bodies in astrocytes while the distribution of extracellular vesicles was altered in mDA and mDA viability was reduced. Culturing the mDA separately showed that these effects were mediated non-cell-autonomously.

Microglia have also been shown to play important roles in neurodevelopment and neurodegeneration, particularly in the case of DA [15, 49, 59]. Unlike astrocytes and neurons, microglia arise from a distinct myeloid lineage, entering the brain from the embryonic yolk sac during early stages of neurodevelopment [43]. Several groups have used microglia and NSC lines in a culture approach to assess the impact of microglia on mDA. Schmidt et al. co-cultured multiple microglial and NSC lines in both direct and indirect format to assess the impact of microglia on mDA development [55]. Microglia enhanced mDA differentiation in both direct and indirect setups. This was verified using two different microglial and three different NSC lines. These findings support *in vivo* evidence of critical roles for microglia in mDA development.

To better recapitulate *in vivo* cellular interactions and cell states for DA, 3D and “2.5D” organoids have been generated [9, 22, 57, 58]. 3D cultures also allow for longer culturing than in 2D [57]. Sozzi et al. developed a protocol that differentiates hPSCs into 3D ventral midbrain organoids [58]. By day 60, these organoids contain mature post-mitotic, neuromelanin + DA and display the molecular profile of human mDA. Lund human mesencephalic (LUHMES) cells—an immortalized cell line—have also been differentiated into 3D “brain spheres” and have been used in assessing chemical toxicity to compounds known to impact DA, such as rotenone [9, 57]. Glial cells, including astrocytes and microglia, have been added to these brain spheres. In addition, a plating step was introduced, giving “2.5D” spheres to allow for assessments of neurite outgrowth. These 3D cultures allow for generation of more mature, longer lasting, DA than in 2D cultures.

One of the most exciting applications for utilization of techniques to differentiate PSCs into different neuronal fates is the use of hiPSCs. Patient-specific hiPSCs can be generated from several cell types, such as fibroblasts, using various approaches, including nuclear reprogramming with viral vectors or microRNAs [1, 34]. This was first achieved for PD patient-derived hiPSCs in 2008 [46]. hiPSCs allow for studying a “disease in a dish”—to study mechanisms and find novel treatments through compound screening. Patient-derived hiPSCs have helped to elucidate pathology and look at biochemical, functional, and morphological phenotypes associated with diseases. These hiPSCs can be studied naively or following a stressor, and be used to examine the impact of gene mutations, such as risk genes for PD or autism spectrum disorders [18, 61, 63, 65]. By following the course of differentiation, different stages of the disease can be assessed. hiPSCs for both sporadic (unknown cause) and familial (known cause) donors are informative. Both have been derived for PD patients—two of the most common with mutations in LRRK2, a kinase risk gene for PD, or SNCA, a protein linked to α-synuclein (αsyn) accumulation, a protein implicated in idiopathic PD. One study used hiPSCs derived from young onset PD and differentiated them into mDA [36]. These mDA showed typical pathology for PD—an accumulation of αSyn and reduction in lysosomes. Application of a lysosomal pathway activator reversed this pathology. An additional advantage of hiPSCs is the window they open into gene-environment interactions (GxE) as will be discussed below.hiPSCs show great promise for translational medicine as they are readily accessible and are patient-specific, allowing for exploration of mechanisms underlying various disorders and diseases [1, 34]. hiPSCs provide additional advantages for screening purposes due to their self-renewal capacity, allowing for expansion of the pool into the necessary number of cells and their pluripotency, allowing for production of most any cell type. Two critical hurdles in the use of hiPSCs can be the heterogeneity of cells derived and the degree of interlaboratory variability and reliability, though cell sorting and phenotyping can be used to purify the cells produced [28].

## Screening

DA have been recognized to play key roles in numerous neurodevelopmental and neurodegenerative disorders. DA have been used in screening approaches to identify the effects of certain chemicals, particularly to identify potential therapeutics for disorders such as PD [2, 34, 45, 50, 61, 65]. These approaches have been used to assess effects on both the initial specification and later maturation phases of DA development. These screens can be used to assess a variety of functional and morphological endpoints—imaging being the most common approach, though functional readouts, including calcium imaging or recording synaptic firing such as with microelectrode arrays, are also possible.

A common method to adapt mDA differentiation protocols for screening applications has been to expand the pool of mDA precursors, or mature mDA, through several passages to create large batches of mDA that can be cryopreserved and subsequently matured into mDA or simply plated and then used in screens [17, 20, 24]. Automated procedures have also been developed. Depending on the desired maturation state, these protocols generally vary from ~ 45–65 days from hPSC specification to mature mDA production.

Perhaps the most widespread use in screening approaches has been with hiPSCs, allowing for assessment of GxE—the crosstalk between genes and one’s physical and social environment and how this may impact phenotype during one’s lifetime. GxE are increasingly thought to contribute to neurodevelopmental and neurodegenerative diseases [33]. This is particularly true for PD as 90–95% of cases are sporadic—cannot be explained purely by genetic effects. Moreover, the DA system is especially sensitive to environmental factors such as metals and pesticides [33].hiPSCs provide an exciting, unique approach to understanding and exploring GxE as they may harbor patient-specific risk genes. These experiments require careful initial phenotyping to characterize differences in the patient-derived hiPSCs as compared to healthy controls. Several screens have been conducted with PD patient-derived hiPSCs carrying common risk genes, differentiating them into mDA. Yamaguchi et al. used hiPSCs containing mutations in either *Parkin* or *PINK1*, two genes that are critical for mitochondrial function [65]. These lines produced the same morphology, marker expression, and induction efficiency as controls, but impaired clearance of mitochondria as well as increased oxidative stress and apoptosis. Yamaguchi et al. employed a 320-compound library to screen for rescuing these deficits in a tiered approach. A primary screen was conducted on the complete library to identify compounds that improved clearance. These hits were then run through further screens for levels of apoptosis and oxidative stress, resulting in a total of four compounds which were then assessed for efficacy in Drosophila and hiPSCs from patients with idiopathic PD. A similar study was conducted by Tabata et al. using the FDA screening library, which consists of a total of 1165 compounds [61]. hiPSCs were derived from patients with mutations in *PARK-2* or *6*, two PD risk genes involved in mitochondrial homeostasis and stress. Patient-derived hiPSCs were screened for chemicals that reduced rotenone-induced apoptosis. Hit chemicals were then pursued with mechanistic studies. hiPSCs harboring mutations in the *SNCA* gene have also been used [2]. These were screened with a small molecule kinase inhibitor library containing 273 compounds. Following a 7-day differentiation to produce NPCs, cells were exposed to individual chemicals and assessed for TH expression normalized to βIII-tubulin for total neurons two weeks later. One hit was identified in this screen and followed up, showing the increase in TH expression to be related to an increase in neurite outgrowth, as well as reduced axonal degeneration, protein aggregation, and phosphorylated αSyn. Moreover, this compound was found to be effective when applied both during the NPC state, day seven, as well as in mature neurons. Protein expression analysis showed upregulation of 118 proteins from this compound.

To our knowledge, no studies have been conducted employing hPSC-derived mDA in toxicity testing for DNT. The only DNT NAMs that do look at DA, and a neuronal subtype in general, employ LUHMES cells. LUHMES have primarily been screened for endpoints of neurite outgrowth and cell viability [19, 62]. In other studies they have been used in toxicity testing focused on single, or a small group of, chemicals to look at a broader range of measures, including metabolism and functional endpoints [37, 54]. As noted above, protocols have been developed to generate 2.5 and 3-D LUHMES spheres with the aim of using these for DNT screening [9, 57]. These spheres, or organoids, allow for greater recapitulation of *in vivo* neuronal development, though may compound analytical demands.

The ReNCell line—an immortalized NPC line derived from the ventral mesencephalon of the human fetal brain, has also been employed for screening—still focused on development, but was geared towards protocol optimization, not DNT NAMs [50]. ReN cells were used in a screening approach to identify compounds that impacted mDA specification or maturation based on doublecortin or TH expression, respectively. From the 5000 compounds, identified hits included statins, TGFβR1 inhibitors, and GSK3β inhibitors. These hits were further assessed for mechanistic information by looking at proliferation, apoptosis, and lineage specification from exposed NPCs. A similar approach could be readily employed for hPSCs.

## Limitations & Gaps in Knowledge

Now that their methodologies are well-established, the time is ripe for PSCs to be utilized in toxicological assessments. *In vitro* NAMs provide numerous advantages in screening for risk assessment, including their increased throughput and reduced costs and animal welfare concerns. This is particularly the case for evaluating the impact of chemicals on development as PSCs provide the best cell-based model of human *in vivo* development, capturing the numerous processes involved in human neurodevelopment, eliminating the need for interspecies extrapolation, and allow for readily employing genetic manipulations. With their pluripotency, PSCs can be utilized to assess the impact of chemical exposure on development in any organ system. Human-specific models are especially advantageous in screening for DNT due to the increased complexity and cell-type specificity of the human brain as compared to the brains of non-human primates and rodents [32, 42]. The field of DNT is thus already realizing the utility of *in vitro* NAMs for risk assessment, helping to fill the dearth of data [21].

To tease apart the complexity of neurodevelopment and explore adverse outcome pathways associated with DNT, the battery consists of numerous processes involved in neurodevelopment, such as proliferation and neurite outgrowth. One major gap in the current DNT battery is assessment of neuronal subtypes. Neuronal subtypes show differential sensitivity to chemical exposures. DA are particularly susceptible to environmental chemicals, which has been linked to the instability and rapid auto-oxidation of DA, increasing the burden of oxidative stress in these cells [25, 39, 55]. In addition, the high ATP-dependency of DA, as well as their gene expression, appear to confer susceptibility [14]. Several studies have looked at the impact of environmentally relevant compounds, such as rotenone, on DA, especially using LUHMES. One of the assays developed for the DNT test battery, the UKN4 assay for neurite outgrowth, uses LUHMES [41]. Screening methods with LUHMES have been developed in 2D, 2.5D, and 3D and provide great promise for toxicity testing [9, 57]. The greater complexity of 3D models better recapitulates *in vivo* cellular interactions, though provides hurdles in performing high-throughput screening as well as understanding mechanisms as compared to 2D assays. As for LUHMES, these cells do not recapitulate human *in vivo* neurodevelopment as they have already been specified down the neuronal lineage and immortalized with a tetracycline-responsive c-myc gene, [57, 66]. Unlike differentiation from PSCs, LUHMES take only ~ six days to generate TH + DA. In addition, while LUHMES display the major hallmarks of DA, they are not fully characterized, including having not been functionally verified via transplantation, and do not represent a defined developmental stage as they express markers of both DA precursors and mature DA [38, 56, 66]. hPSC-derived mDA provide greater recapitulation of human *in vivo* development.hiPSC-derived mDA have been used in compound screening approaches aimed primarily at identifying potential drug candidates for neurodegenerative diseases [61, 65]. While these studies have shown promise for studying neurodegenerative diseases such as PD, we caution that many protocols for DA neuron differentiation do not capture mature DA—only rarely have these cultures been assessed for neuromelanin release, which is one of the final steps for DA neuron maturation, let alone characterized as neurons in the aging brain [58]. Criteria to distinguish neurodevelopmental-, versus neurodegenerative-relevant mDA have not been established. Strategies aimed at either lifestage will be limited until this delineation has been clarified. One avenue that provides promise to be able to distinguish between neurodevelopmental and neurodegenerative mDA is the use of hiPSCs. Human dermal fibroblasts can be used to derive DA either indirectly via hiPSCs, or directly through transduction with reprogramming factors, to produce induced DA. The derived DA are distinct in that the induced DA reflect the epigenetics, transcriptomics, and oxidative stress of their host, whereas the hiPSC-derived DA do not reflect this aging signature [23]. While hiPSCs provide great potential for translational relevance, the differentiation capacity of of hiPSCs can vary widely, even from the same tissue of origin, which may necessitate careful phenotyping [28].

More generally, there is still a lack of knowledge on the programs that drive development of DA *in vivo* and a need for further optimization of mDA protocols. The lack of consistency in methods to derive mDA impedes cross-study comparisons. Studies should provide all necessary details to enable easy replication. These include chemical sourcing and purity, substrate, including substrate concentration, the total days *in vitro* at which assays and procedures are performed, seeding density, frequency of feeding, relative expression levels of genes critical to DA neuron development, including FOXA2, LMX1A, TH, and VMAT2, and the proportion of DA in assayed cultures. Studies should also perform assessments of additional neuronal subtypes that cells may have matured into, including astrocytes and cholinergic, serotonergic, and noradrenergic neurons. For studies exploring questions of neurodegeneration, additional functional assessments are crucial.

Based on the high degree of cell death and often low proportion of TH + neurons produced, protocols for DA neuron development could be improved. The FP approach developed by Studer utilizes knockout serum and results in a high degree of cell death, calling for further optimization [60]. In our hands, the use of defined media instead of knockout serum, reduces the degree of cell death. While most protocols rely on SHH, FGF8, and WNT signaling, more recent studies have shown an important role for BMP signaling [29]. Addition of BMP5 and BMP7 has been shown to increase the yield of mDA *in vitro*. Other signaling pathways may also be involved, such as DKK [8]. Moreover, the difference in *in vivo* development of distinct mDA subtypes is still poorly understood. This could be critical to understanding of, and treatment strategies for, disorders such as PD, in which A9 neurons of the SNpc are particularly vulnerable [8, 40]. Further studies are needed to clarify the development of DA *in vivo*. Meanwhile, publication of all experimental details will help to improve study replicability.

## Conclusion

PSCs are beginning to be utilized in toxicology and provide an opportune approach for toxicant screening of neuronal subtypes—a critical component of the DNT NAM battery that is currently lacking. As mDA development is now well understood, PSC-derived mDA could readily be used to identify compounds that interfere with DA development. PSC screening tools, in addition to utilizing patient-specific hiPSCs, could help to elucidate the role of DA in various neurodevelopmental and neurodegenerative disorders. Similar approaches could be developed for other neuronal subtypes. Such screening approaches would be a great contributor to the current DNT NAM battery to tease apart the differential sensitivity of each subtype to environmental chemicals during development.

## Figures and Tables

**Table 1:** Signaling factors & markers used for DA differentiation

AADC	Aromatic L-amino acid decarboxylase; enzyme for DA synthesis
ASCL1	bHLH transcription factor for neuronal commitment & differentiation, transduction with Nurr1 & GDNF increases DA yield, proneural
CHIR99021	GSK3β inhibitor, activates WNT signaling
CORIN	Cell surface marker in midbrain FP, marker of midline progenitors
DAT	Na/Cl dependent neurotransmitter family; expressed once migrate
EN1	HOX gene involved in pattern formation, expressed in midbrain FP by ~ E8 in mouse
FGF8	Growth factor secreted by hindbrain, helps induce mDA fate
FOXA2	FP marker, also expressed in human, expression remains; induces Neurogenin2, then Nurr1 & EN1; transduction w/ Nurr1 results in increased midbrain specificity
LDN193189	Small molecule inhibitor of BMP signaling for dual SMAD inhibition (dSMADi)
LMX1A	FP marker, also expressed in human, stable expression following induction; expressed more lateral than FOXA2, also somewhat basal
Neurogenin2	Expressed after neurogenesis, proneural; “Ngn2”
Noggin	Binds to & inhibits BMP signaling
Nurr1	Transduction alone or w/ FOXA2, Ngn2, ASCL1, or PITX3 induces mDA that can engraft in vivo; expressed by postmitotic neuroblasts, not midbrain specific; necessary for mDA, expressed after FOXA2, prior to TH & PITX3, expression maintained through differentiation, activates TH promoter, needed for DAT & VMAT induction, suggested to regulate PITX3
OTX2	HOX transcription factor for head development & patterning; regulates mDA neurogenesis, expressed in midbrain FP by day 11
PITX3	Transcription factor expressed once DA progenitors migrate, addition of improves mDA production efficiency
Purmorphamine	Potent small molecule activator of SHH
SB431542	Inhibits TGFβ signaling, which subsequently blocks nodal, activin; for dSMADi
SHH	Sonic hedgehog (SHH), helps induce mDA fate
TH	Tyrosine hydroxylase (TH), expressed once migrate, expressed by all catecholaminergic lineages
VMAT2	Transmembrane protein for monoamine transport; expressed once migrate
WNT1	Secreted by midbrain side, needed for induction of FP cells to DA

## Abbreviations

DA: Dopaminergic neurons
DAT: DA active transporter
DNT: Developmental neurotoxicity
dSMADi: Dual SMAD inhibition
ESCs: Embryonic stem cells
FP: Floor plate
GW: Gestation weeks
GxE: Gene-environment interactions
hiPSC: Human induced pluripotent stem cells
hPSCs: Human pluripotent stem cells
iPSCs: Induced pluripotent stem cells
LUHMES: Lund human mesencephalic cells
mDA: Midbrain DA neurons
NAMs: New approach methodologies
NPCs: Neural precursor cells
PD: Parkinson’s Disease
PSCs: Pluripotent stem cells
RRF: Retrorubral field
SHH: Sonic hedgehog
SNpc: Substantia nigra pars compacta
TH: Tyrosine hydroxylase
VMAT2: Vesicular monoamine transporter
VTA: Ventrotegmental area
